# Targeted muscle reinnervation attenuates neuropathic pain and neuroma development in a rat model of tibial nerve transection

**DOI:** 10.3389/fbioe.2026.1758496

**Published:** 2026-02-27

**Authors:** Li Li, Ainizier Yalikun, QiYue Zhang, DeBin Xiong, Tao Jiang, Fan Bu, QingTang Zhu, Aihemaitijiang Yusufu

**Affiliations:** 1 Department of Microrepair and Reconstruction, The First Affiliated Hospital of Xinjiang Medical University, Urumqi, Xinjiang, China; 2 Department of Orthopedics, The Fourth Affiliated Hospital of Xinjiang Medical University, Urumqi, Xinjiang, China; 3 Animal Center, Xinjiang Medical University, Urumqi, Xinjiang, China; 4 Department of Orthopedics, Hetian Regional People’s Hospital, Hetian, Xinjiang, China; 5 Department of Orthopedics and Microsurgery, First Affiliated Hospital of Sun Yat-sen University, Guangzhou, Guangdong, China

**Keywords:** nerve transection, neuroma prevention, neuropathic pain, rat model, targeted muscle reinnervation

## Abstract

**Background:**

Peripheral nerve injuries often lead to painful neuroma formation and chronic neuropathic pain, and the optimal surgical strategy for prevention remains debated. Targeted muscle reinnervation (TMR), regenerative peripheral nerve interfaces (RPNI), and nerve-in-muscle implantation (NIM) are surgical techniques developed to mitigate neuroma-related pain, but their relative efficacy has not been compared systematically. This preclinical study compared TMR, NIM, and two RPNI variants in a rat tibial nerve transection model to identify which approach best reduces neuroma formation and pain.

**Methods:**

Sprague-Dawley rats underwent right tibial nerve transection and were randomized into five groups: control (no repair), NIM, W-RPNI (wrapped RPNI), E-RPNI (embedded RPNI), or TMR. Behavioral outcomes including gait analysis (CatWalk), mechanical hypersensitivity (von Frey test), thermal hyperalgesia (Hargreaves test), and neuroma tenderness were assessed over 12 weeks. At week 12, distal nerve stumps and L4–L5 dorsal root ganglia (DRG) were harvested for histological evaluation, immunohistochemistry/immunofluorescence, and molecular analyses (qRT-PCR and Western blot) targeting pain- and inflammation-related biomarkers.

**Results:**

By 12 weeks, TMR-treated rats showed the most robust improvements, including significantly longer stance duration, larger paw contact area, near-baseline withdrawal thresholds, and minimal neuroma tenderness, whereas untreated controls developed gross neuromas and persistent hypersensitivity. TMR also preserved organized nerve architecture with orderly axonal regeneration and minimal collagen I/III fibrosis at the stump. Molecular assays confirmed that TMR markedly attenuated nociceptive and inflammatory signaling, with TMR rats exhibiting the lowest expression of pain-related mediators (c-Fos, TRPA1, TRPV1, CGRP, NPY, BDNF) and pro-inflammatory/fibrotic markers (galectin, α-SMA, IL-1β, TNF-α, TGF-β) in nerve and DRG tissues. Conversely, the anti-inflammatory cytokine IL-10 and axonal ion pump subunits ATP1A2/ATP2B1 were significantly upregulated with TMR. Outcomes for the two RPNI groups were similar to each other and generally intermediate between TMR and control.

**Conclusion:**

TMR was superior to RPNI variants and NIM in preventing neuroma formation and alleviating neuropathic pain in this animal model. These findings support TMR as a promising surgical strategy to mitigate post-amputation neuroma pain.

## Graphical Abstract

**Figure:**
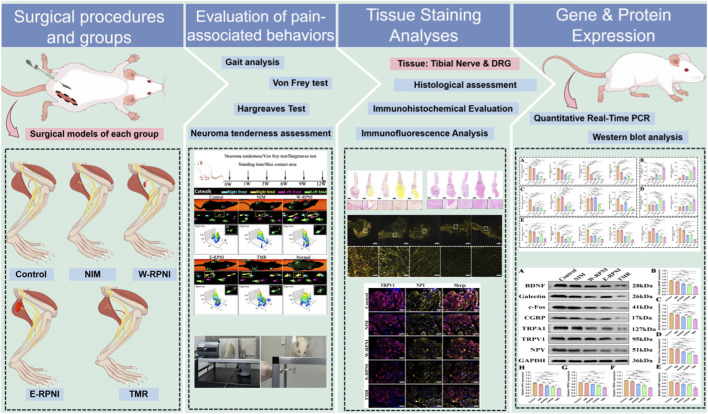

## 1 Introduction

Peripheral nerve injury and limb amputation frequently result in painful neuroma formation, a major cause of chronic neuropathic pain. In the United States, nearly 185,000 amputations occur annually, leaving close to two million individuals with residual limbs. Up to one-quarter of these patients develop chronic pain due to symptomatic neuromas or phantom limb pain (Bowen et al., 2017). Neuromas consist of disorganized axons intertwined with scar tissue, producing focal, debilitating pain that interferes with prosthesis use (Anantavorasakul et al., 2020). This aberrant regenerative process, together with central changes underlying phantom limb pain, imposes significant physical and psychological morbidity (Culp and Abdi, 2022). Traditional pharmacological, psychological, and surgical treatments—such as neuroma excision or nerve burial—have yielded inconsistent results, underscoring the urgent need for more effective preventive strategies (Yang et al., 2023).

Over recent decades, multiple surgical strategies have been developed to guide regenerating axons and reduce neuroma-related and post-amputation pain. These include traction neurectomy (van den Berg et al., 2025), implantation of the nerve stump into muscle (Wang et al., 2023) or bone (Karczewski et al., 2022), biomaterial-based capping approaches (Borcherding et al., 2025), and regenerative peripheral nerve interfaces (RPNI) (Kubiak et al., 2021). While these techniques have shown potential benefits, reported outcomes vary across studies and robust head-to-head comparative evidence remains limited. Clinically, RPNI has been associated with improved neuroma-related pain outcomes, while phantom limb pain may still persist in a substantial proportion of patients (e.g., ∼51% in one cohort) (Kubiak et al., 2022). By contrast, targeted muscle reinnervation (TMR) provides regenerating axons with functional motor endplates. In rodent models, TMR has been shown to redirect axonal growth toward the muscle target and is associated with a more organized regeneration pattern and less disorganized axonal sprouting/neuroma-like axonal tangling (Senger et al., 2023). Clinical studies demonstrate that TMR reduces residual and phantom limb pain and lowers neuroma recurrence compared with conventional excision (Valerio et al., 2019; Valerio et al., 2021; Keen et al., 2024). Nevertheless, mechanistic understanding of how TMR compares with RPNI variants or nerve-in-muscle implantation in modulating peripheral and central nociceptive pathways remains incomplete, as systematic cross-comparisons are lacking.

To address this knowledge gap, we conducted a comprehensive preclinical study using a rat tibial nerve transection model to directly compare the efficacy of TMR, nerve-in-muscle implantation (NIM), and two RPNI variants (wrapped and embedded). Our primary objective was to test whether coapting the transected tibial nerve to a motor branch of the biceps femoris muscle offers superior functional recovery and pain relief compared with burying or wrapping the nerve in muscle grafts. We evaluated gait patterns, mechanical and thermal sensitivity, and neuroma tenderness over a 12-week period. Histological organization and collagen deposition were examined using hematoxylin and eosin, Masson’s trichrome, and Sirius red staining. Immunohistochemical and immunofluorescent analyses targeted neuronal activation (c-Fos), pain neuropeptides (substance P, CGRP), fibrosis (α-SMA), and neurotrophic and inflammatory mediators (BDNF, galectin-3, TRPA1, TRPV1, NPY). qRT-PCR further assessed pain-, inflammatory-, and fibrosis-related gene expression in tibial nerves and dorsal root ganglia. We hypothesized that TMR would not only improve behavioral outcomes but also suppress nociceptive and inflammatory signaling while upregulating antinociceptive and anti-inflammatory pathways.

In the following sections, we present our surgical procedures and experimental design, report behavioral, histological, and molecular outcomes, and discuss how these findings advance understanding of neuroma pathophysiology and inform nerve transfer strategies. By integrating functional assessments with detailed cellular and molecular analyses, this work aims to clarify how targeted reinnervation shapes the peripheral microenvironment and central sensitization to achieve durable analgesia.

## 2 Method and material

### 2.1 Surgical procedures and groups

Male Sprague-Dawley rats (200–230 g) were obtained from the Experimental Animal Center of Xinjiang Medical University. After screening, animals were housed under constant temperature and humidity with a 12 h light/dark cycle. For surgery, rats were anesthetized with an intraperitoneal injection of 2% sodium pentobarbital (40 mg/kg). Under aseptic conditions, a skin incision was made along the lateral aspect of the right thigh. The underlying muscles were bluntly separated to expose the sciatic nerve, and the tibial nerve was dissected under a surgical microscope and transected to establish a tibial nerve transection model. Animals were randomly assigned to five groups (n = 12 per group; total 60 rats); Control group: the tibial nerve was sharply transected; the proximal stump was ligated with 10-0 nylon suture, and 10 mm of the distal stump was excised to prevent spontaneous reconnection/regeneration. NIM group:proximal stump of the transected tibial nerve was sutured to the belly of the biceps femoris muscle. W-RPNI (Wrapped-RPNI) group: proximal tibial nerve stump was wrapped with a small piece of muscle graft harvested from the contralateral extensor digitorum longus muscle (only a small segment of the contralateral extensor digitorum longus muscle was harvested, rather than excising the entire EDL). E-RPNI (Embedded -RPNI) group: based on the W-RPNI procedure, the muscle wrap was further inserted into an adjacent innervated portion of the biceps femoris muscle. TMR group: after tibial nerve transection, a motor branch of the adjacent biceps femoris muscle was isolated and transected, and the distal stump of this branch was coapted to the proximal stump of the tibial nerve. For the neurorrhaphy, the biceps femoris motor branch coaptation was performed under an operating microscope using fine monofilament nylon sutures (11-0) to achieve a tension-free epineurial repair. Following established rodent TMR protocols, the target motor branch was transected approximately 1 cm proximal to its entry into the biceps femoris muscle, and the proximal tibial nerve stump was coapted to the distal motor branch at this level. Preserving this short distal segment maintains a viable Schwann-cell–containing scaffold for regenerating axons and allows for a secure, tension-free neurorrhaphy. The muscle and skin layers were closed with 5-0 silk sutures. All rats received an intramuscular injection of penicillin (800,000 units) to prevent postoperative infection. Behavioral assessments were conducted 1 day before surgery and at 1, 3, 6, 9, and 12 weeks postoperatively. At 12 weeks, histological and molecular analyses were performed to evaluate neuroma formation, pain behaviors, and the inflammatory microenvironment at the nerve stump (five biological replicates per group) (See in Supplementary Figure S1).

### 2.2 Evaluation of pain-associated behaviors

Evaluation of pain-associated behaviors One day prior to behavioral testing, rats designated for pain-related behavioral assessments were housed individually to minimize environmental stress. On the test day, all animals were placed on the designated testing platform for a 1-h acclimation period. All behavioral tests were performed by a single investigator blinded to the experimental group assignments. Behavioral measurements were conducted at baseline (the day before surgery) and at 1, 3, 6, 9, and 12 weeks postoperatively.

#### 2.2.1 Gait analysis

Given that neuropathic pain is closely associated with alterations in locomotor patterns, gait analysis was adopted as an indirect measure of pain. Dynamic changes in gait parameters were monitored at each designated time point. Specifically, the stand phase duration and maximal contact area of the affected hind limb were recorded using the CatWalk gait analysis system and compared with baseline values.

#### 2.2.2 Von Frey test

Mechanical sensitivity of the affected hind paw was quantified using a calibrated set of Von Frey filaments. Von Frey testing was performed on the plantar surface within the territory innervated by the tibial nerve. Filaments were applied perpendicularly to the skin surface with sufficient force to induce slight bending, maintaining contact for 2–3 s. Testing began with the thinnest filament; if no response was observed, progressively thicker filaments were applied. Nociceptive withdrawal responses, including vocalization with rapid paw withdrawal, flicking, and/or licking of the hindlimb, were recorded when elicited by any of the filament stimulations (Yousuf et al., 2020; Li et al., 2020).

#### 2.2.3 Hargreaves test

Hargreaves test Thermal hyperalgesia was assessed by measuring the paw withdrawal latency in response to a radiant heat source (infrared beam) applied to the lateral plantar surface of the affected (right) hind paw. A motion sensor automatically recorded the withdrawal latency. A cut-off time of 15 s was set to prevent potential paw tissue damage or burn injury (Zhao et al., 2024; Jackson et al., 1995; Mak et al., 2021).

#### 2.2.4 Neuroma tenderness assessment

Neuroma tenderness was evaluated using a protocol adapted from the Tinel-like test. In each trial, the neuroma site was gently tapped five times with a single von Frey filament (15 g). Five consecutive trials were performed per animal, and the percentage of trials showing a pain response—consistent with the criteria described in the von Frey test—was calculated as an index of neuroma tenderness.

### 2.3 Histological assessment

At 12 weeks postoperatively, rats from each group were anesthetized, and the original surgical site was re-exposed by careful dissection to harvest the treated tibial nerve. In the NIM and both RPNI groups, the tibial nerve together with the distal muscle tissue was excised, whereas in the TMR group, the tibial nerve along with the distal motor branch of the biceps femoris muscle and its innervated muscle portion was removed. All harvested specimens were processed for serial sectioning. Hematoxylin and eosin (H&E) staining was performed to assess whether the structure and growth of the distal nerve stump after intervention were orderly. Masson’s trichrome staining and Sirius red staining were used to evaluate collagen formation and distribution in the distal segment of the nerves in each group. For sample processing, the specimens were fixed in 4% paraformaldehyde, embedded in paraffin (Leica, Germany), and sectioned into 5-μm-thick slices using a microtome (Leica, Germany). The stained sections were observed under an optical microscope; for Sirius red staining, polarized light microscopy (Nikon, Japan) was additionally employed for imaging.

### 2.4 Immunohistochemical evaluation

Dorsal root ganglia (DRG) from the L4-L5 spinal segments and corresponding ipsilateral tibial nerve specimens collected after surgical intervention were processed as described above. Paraffin sections were deparaffinized, rehydrated, and subjected to antigen retrieval, followed by blocking with 5% normal goat serum for 30 min at room temperature. For tibial nerve sections, primary antibody incubation was performed overnight at 4 °C using anti-cellular proto-oncogene Fos (c-Fos) antibody (Rabbit, 1:100, Affinity, AF5354), anti-Brain-Derived Neurotrophic Factor (BDNF) antibody (Rabbit, 1:800, Affinity, DF6387), and anti-Galectin antibody (Rabbit, 1:400, Proteintech, 14979-1-AP). After three rinses in PBS (pH 7.4), sections were incubated for 50 min at room temperature with HRP-conjugated goat anti-rabbit IgG (H+L) secondary antibody (1:1000, Sino Biological, SSA004). Immunoreactivity was visualized using 3,3′-diaminobenzidine (DAB) substrate solution (ZSGB-Bio, ZLI-9018) under microscopic observation. For DRG sections, primary antibody incubation was similarly performed overnight at 4 °C with anti-c-Fos (Rabbit, 1:100, Affinity, AF5354), anti-α-Smooth Muscle Actin (α-SMA) antibody (Rabbit, 1:3000, Proteintech, 14395-1-AP), and anti-Substance P antibody (Rabbit, 1:100, Bioss, bs-0065R). After three washes in PBS, sections were incubated with HRP-conjugated goat anti-rabbit IgG (H+L) secondary antibody (1:1000, Sino Biological, SSA004) for 50 min at room temperature. DAB substrate solution (ZSGB-Bio, ZLI-9018) was then applied until optimal staining was achieved. All sections were dehydrated through a graded ethanol series, cleared, and mounted with neutral balsam mounting medium (Solarbio, G8590). Quantitative analysis of immunostaining was performed using Image-Pro Plus software (version 6.0; Media Cybernetics, Inc., USA).

### 2.5 Immunofluorescence analysis

#### 2.5.1 Immunofluorescence analysis

DRG from the L4–L5 spinal segments and ipsilateral tibial nerve specimens were processed as described above. Paraffin sections were deparaffinized in xylene, rehydrated through a graded ethanol series, and subjected to antigen retrieval in EDTA buffer (pH 9.0). After blocking with 5% normal goat serum for 30 min at room temperature, double-label immunofluorescence was performed using the following antibody pairs on separate sections, and sections were incubated overnight at 4 °C with the corresponding primary antibodies.

(1) CGRP/TRPA1 (tibial nerve and DRG sections): Anti-Calcitonin Gene-Related Peptide (CGRP) antibody (Goat, 1:200, Thermo Fisher Scientific, PA1-85250) and anti-Transient Receptor Potential Ankyrin 1 (TRPA1) antibody (Rabbit, 1:200, Affinity Biosciences, DF13269). After three washes with PBS (pH 7.4), sections were incubated for 50 min in the dark at room temperature with FITC-conjugated donkey anti-goat IgG (H+L) (1:500, Bioss, bs-0294D-FITC) and Alexa Fluor 594-conjugated goat anti-rabbit IgG (H+L) (1:200, Bioss, bs-0295G-AF594) secondary antibodies. Nuclei were counterstained with DAPI-containing mounting medium (Bioss, C02-04002). (2) CD3/CD68 (tibial nerve sections, on separate sections from CGRP/TRPA1): anti-CD3 antibody (Mouse, 1:200, Bioss, bsm-54036M) and anti-CD68 antibody (Rabbit, 1:200, Bioss, bs-1432R). After PBS rinses, sections were incubated in the dark with Cy3-conjugated AffiniPure goat anti-mouse IgG (H+L) (1:500, Proteintech, SA00009-1) and BF647-conjugated goat anti-rabbit IgG (H+L) (1:500, Bioss, bs-0295G-BF647) secondary antibodies, followed by nuclear counterstaining with DAPI-containing mounting medium. (3) TRPV1/NPY (DRG sections, on separate sections from CGRP/TRPA1): anti-Transient Receptor Potential Vanilloid 1 (TRPV1) antibody (Mouse, 1:500, Abcam, ab203103) and anti-Neuropeptide Y (NPY) antibody (Rabbit, 1:500, Affinity Biosciences, DF6431). After PBS rinses, sections were incubated in the dark with CoraLite647-conjugated F(ab')2 fragment goat anti-mouse IgG (H+L) (1:500, Proteintech, SA00014-10) and Cy3-conjugated AffiniPure goat anti-rabbit IgG (H+L) (1:500, Proteintech, SA00009-2) secondary antibodies, followed by nuclear counterstaining with DAPI-containing mounting medium. Images were acquired using an upright fluorescence microscope (Nikon Eclipse C1, Japan) under identical exposure settings across all groups. Quantitative analysis of fluorescence intensity and positively stained area was performed using Image-Pro Plus software (version 6.0; Media Cybernetics, Inc., USA) by an investigator blinded to the experimental group assignments.

### 2.6 Quantitative real-time PCR

Total RNA was extracted from tibial nerve specimens (n = 15, 5 groups, 3 biological replicates) and dorsal root ganglia (DRG) specimens (n = 15, 5 groups, 3 biological replicates) using RNAiso Plus reagent (Takara bio, 9109). RNA quantity and purity were assessed with a microvolume spectrophotometer (UNano-1000, Youmi, China) by A260/A280 ratio measurement. One microgram of RNA from each sample was reverse transcribed into cDNA using the RT Easy™ II Kit (Foregene, RT-01022/01023). Gene-specific primers were designed from NCBI sequences and synthesized by Tsingke Biotechnology (China), with sequences listed in Supplementary Table S1. GAPDH served as the internal control. Target genes for tibial nerve included APOE, CSF, TRPV1, CGRP, ATP2b1, ATP1a2, CCL2, α-SMA, IL-1β, TNF-α, TGF-β, and IL-10; for DRG, the targets were Bdnf, c-Fos, Galectin, NPY, TRPV1, and CGRP. Real-time PCR was performed using SYBR qPCR SuperMix Plus (Lanyun Biotechnology, M00041) on a SLAN-96S Real-Time PCR System (Shanghai Hongshi Medical Technology). Each 10 µL reaction contained 5 µL SYBR Green mix, 0.5 µL of each primer (10 µM), and 4 µL cDNA. The program consisted of 95 °C for 2 min, followed by 39 cycles of 95 °C for 15 s and 58 °C for 30 s, with a melt curve from 60 °C to 95 °C.All reactions were run in triplicate with no-template controls. Relative expression was calculated using the 2^−ΔΔCT^ method and normalized to GAPDH. Values are presented as mean ± standard deviation (SD).

### 2.7 Western blot analysis

Total protein was extracted from the ipsilateral DRG specimens using RIPA lysis buffer (Solarbio, R0010) supplemented with phenylmethylsulfonyl fluoride (PMSF, 1:100). Protein concentrations were determined with a bicinchoninic acid (BCA) protein assay kit (Solarbio, PC0020). Equal amounts of protein (20–40 µg) were separated on 6%–15% SDS-PAGE gels and transferred onto polyvinylidene difluoride (PVDF) membranes (0.22µm, Merck Millipore, ISEQ00010) using wet transfer. Membranes were blocked with 3% bovine serum albumin (BSA, Biosharp, 22299019) in TBST for 90 min at 37 °C and incubated overnight at 4 °C with the following primary antibodies: anti-GAPDH (Mouse, 1:5000, Zen Bioscience, 200306-7E4), anti-CGRP (Rabbit, 1:500, Huabio, HA722991), anti-BDNF (Rabbit, 1:2000, Huabio, ET1606-42), anti-c-Fos (Rabbit, 1:500, Huabio, ET1701-95), anti-Galectin (Rabbit, 1:1000, Abcam, ab76245), anti-NPY (Rabbit, 1:500, Huabio, HA720061), anti-TRPV1 (Rabbit, 1:1000, Abcam, ab305299), and anti-TRPA1 (Rabbit, 1:500, Huabio, ER1803-91). After TBST washes, membranes were incubated with horseradish peroxidase (HRP)-conjugated goat anti-mouse or goat anti-rabbit IgG secondary antibodies (1:10,000, Zen Bioscience, 511103) for 1 h at room temperature. Immunoreactive bands were visualized using an ECL substrate kit (Lanyun Biotechnology, W00091) and imaged with a gel documentation system. Band intensities were quantified using Image-Pro Plus software and normalized to GAPDH.

### 2.8 Statistical analyses

Data are presented as the mean ± standard error of the mean (SEM). Statistical evaluations were carried out using GraphPad Prism, version 10.0 (GraphPad Software Inc., California, USA). Comparisons between two independent groups were performed with the unpaired Student’s t-test. For analyses involving more than two groups, one-way analysis of variance (ANOVA) followed by Tukey’s multiple comparison *post hoc* test was applied for selected pairwise comparisons. Statistical significance was indicated using the following notation: *p < 0.05, **p < 0.01, ***p < 0.001, and ****p < 0.0001; values below these thresholds were regarded as statistically significant. All reported quantitative data were derived from a minimum of three independent experimental replicates.

## 3 Results

### 3.1 Behavioral assessment of neuropathic pain

**FIGURE 1:**
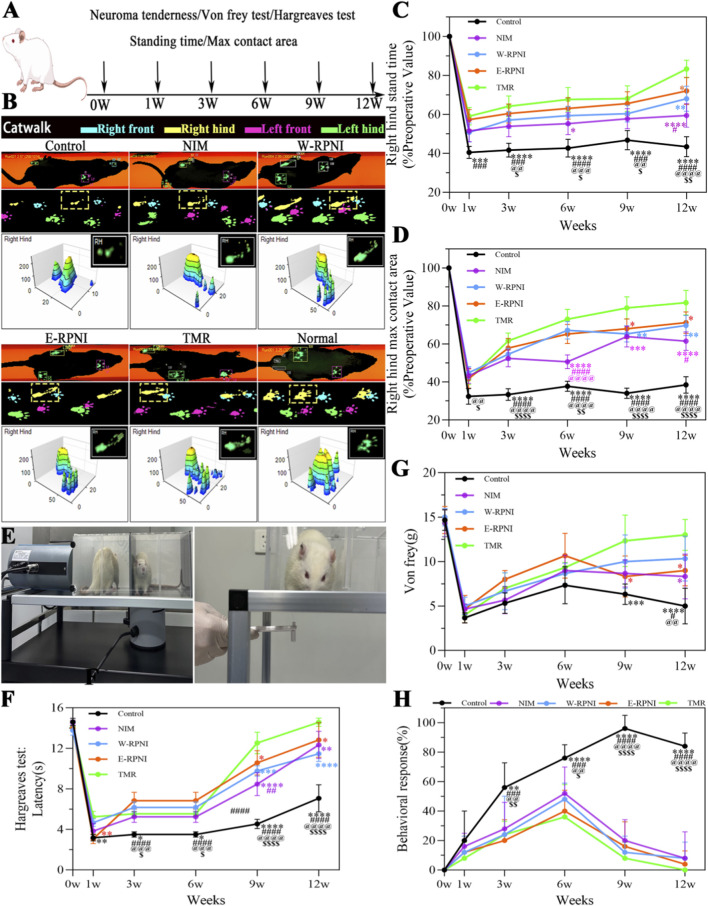
Comprehensive behavioral assessment of neuropathic pain and locomotor function following surgery. **(A)** Schematic illustrating the evaluation of pain-related behaviors at multiple time points. **(B)** Representative gait footprints over a 3-second period and corresponding intensity map of the left hind paw at 12 weeks post-surgery. **(C)** Proportion of stand phase duration at various postoperative time points within a 5-second window. **(D)** Proportion of maximal paw contact area at various postoperative time points within a 5-second window. **(E)** Demonstration of procedures for the von Frey test and Hargreaves test. **(F)** Comparison of von Frey test results among groups at 12 weeks post-surgery. **(G)** Comparison of Hargreaves test results among groups at 12 weeks post-surgery. **(H)** Comparison of neuroma tenderness assessment results among groups at 12 weeks post-surgery. Data are expressed as means ± SD. Symbols: *p < 0.05 vs. TMR group; ^#^ *p* < 0.05 vs. E-RPNI group; ^@^ *p* < 0.05 vs. W-RPNI group; ^$^ *p* < 0.05 vs. NIM group. n = 5 for all groups.

To evaluate the analgesic effects of TMR relative to other interventions, pain-related behavioral assessments were conducted at baseline (the day before surgery) and at 1, 3, 6, 9, and 12 weeks postoperatively (Figure 1A). CatWalk gait analysis (Figure 1B) revealed that during the first 9 weeks, a significant difference in standing time was detected only in the NIM and TMR groups at week 6 compared with the control group. By week 12, the TMR group exhibited a markedly longer standing time than all other groups (Figure 1C). Analysis of maximal contact area showed that from week 6 onwards, all three intervention groups differed significantly from the NIM group; at week 12, the TMR group demonstrated the greatest improvement relative to all groups, whereas no significant difference was observed between E-RPNI and W-RPNI for either gait parameter (Figure 1D). Mechanical hypersensitivity assessed using the von Frey test (Figure 1E), showed no significant differences among groups during the first 6 weeks postoperatively, although all groups exhibited a pronounced reduction in withdrawal thresholds compared with baseline. At weeks 9 and 12, both TMR and E-RPNI achieved significantly greater recovery from mechanical hypersensitivity than the other groups (Figure 1F). Thermal hyperalgesia, evaluated via the Hargreaves test, improved significantly in the TMR group from week 9 onward, reaching near-baseline values by week 12 (Figure 1G). Neuroma tenderness assessment indicated that, except for the control group, all interventions produced a gradual reduction in localized pain at the neuroma site starting from week 6 after surgery. Although these intergroup differences did not reach statistical significance, the TMR group consistently showed the most favorable tenderness scores (Figure 1H). Overall, these findings suggest that TMR not only significantly alleviates abnormal pain in the neuroma region but also enhances recovery from mechanical hypersensitivity and thermal hyperalgesia during the course of neuroma development after nerve transection.

### 3.2 Histomorphometric analysis of distal nerve segments

**FIGURE 2:**
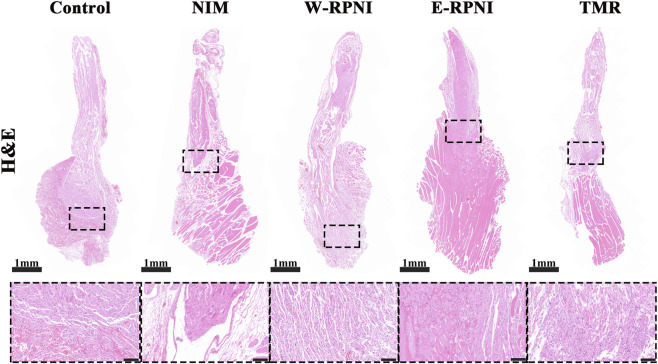
H&E staining of longitudinal sections of the distal segment of the tibial nerve in each group, with corresponding locally enlarged views. Scale bar = 1 mm; magnified view, scale bar = 100 μm.

At 12 weeks postoperatively, classical spheroid neuromas were consistently observed at the distal tibial nerve stumps in the control group (See in Supplementary Figure S1). In contrast, no gross neuroma formation was detected in the other intervention groups. In the TMR group, the coaptation site between the tibial nerve and the motor branch of the biceps femoris maintained intact nerve continuity without evidence of neuroma-like enlargement. Histological analyses were performed on the entire harvested segment, including the distal tibial nerve stump and the associated coapted muscle or nerve tissue. Representative H&E images are shown in Figure 2. In the control group, high-magnification views of the distal tibial nerve revealed hallmark features of neuroma formation: disorganized cellular proliferation, irregular axonal regeneration, muscle infiltration, and patchy or fascicular arrangements of myelinated fibers. In the NIM group, although the distal tibial nerve stump was enclosed by muscle tissue, it maintained a clear separation, with relatively few nerve fibers projecting into the surrounding muscle when compared with the W-RPNI and E-RPNI groups. In the E-RPNI group, the distal stump-muscle boundary appeared poorly defined, with numerous branching nerve fibers infiltrating deeply into the wrapped muscle tissue. Notably, in the TMR group, the coaptation site between the tibial nerve and the motor branch of the biceps femoris exhibited highly organized nerve regeneration, with no evidence of aberrant axonal proliferation; even at the anastomosis line, the tissue architecture remained orderly.

**FIGURE 3:**
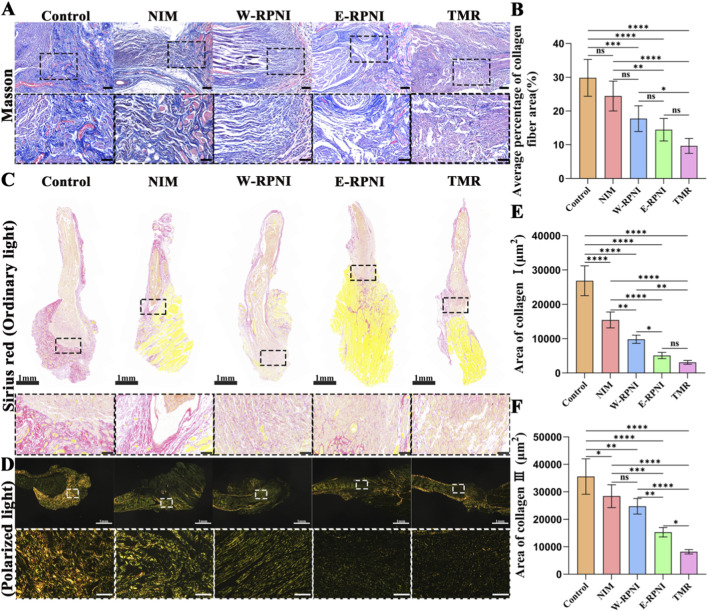
Histological evaluation of the distal segment of the tibial nerve. **(A)** Masson’s trichrome staining of longitudinal sections of the distal tibial nerve. Scale bar = 200 μm; magnified view, scale bar = 100 µm. **(B)** Quantitative analysis of the percentage area of collagen fibers in each group. **(C)** Bright-field images of Sirius red-stained longitudinal sections of the distal tibial nerve. Scale bar = 1mm; magnified view, scale bar = 100 µm. **(D)** Polarized light images of Sirius red-stained longitudinal sections of the distal tibial nerve. Scale bar = 1 mm; magnified view, scale bar = 100 µm. **(E,F)** Quantitative analysis of the mean area of type I and type III collagen fibers in each group. Data are expressed as means ± SD,*p < 0.05, **p < 0.01, ***p < 0.001, ****p < 0.0001, ns: no significance. n = 5 for all groups.

Excessive fibrosis is recognized as the second major driver of neuroma-associated pain (Dong et al., 2023). During fibrotic progression, regenerating axons may be entrapped by myofibroblasts, resulting in persistent mechanical irritation. Masson’s trichrome staining showed that in the control group, distal nerve stumps contained densely packed blue-stained collagen intermingled with numerous small, scattered nerve fascicles arranged in a highly disorganized pattern (Figure 3A). In contrast, the TMR group displayed a well-aligned nerve fiber architecture at the coaptation site, with only mild collagen deposition. Although the collagen fiber area percentage in the TMR group was comparable to that in the E-RPNI group, it was lower than in all other groups (Figure 3B). Sirius Red staining under polarized light differentiated collagen subtypes: type I collagen appeared yellow-orange and type III collagen appeared green (Figures 3C,D). At 12 weeks postoperatively, type I collagen content was lowest in the TMR group, similar to the E-RPNI group, but markedly lower than in all other groups (Figure 3E). Type III collagen levels followed the same trend, with the TMR group showing the lowest expression and significant reductions compared to all other groups (Figure 3F). Together, these histological and quantitative data demonstrate that TMR not only prevents the development of painful neuromas but also attenuates excessive deposition of both type I and type III collagen associated with fibrotic overgrowth.

### 3.3 Immunohistochemical findings

**FIGURE 4:**
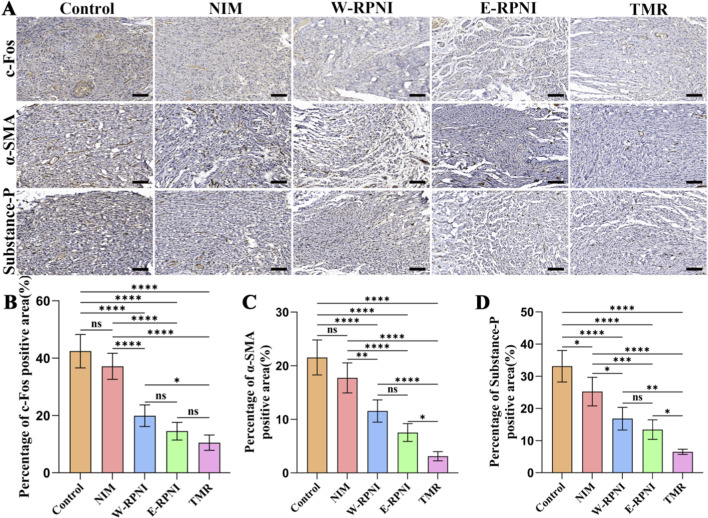
Immunohistochemical analysis of the distal segment of the tibial nerve. **(A)** Immunohistochemical expression of c-Fos, α-SMA, and Substance-P in longitudinal sections of the distal tibial nerve from each group. Scale bar = 100 μm. **(B–D)** Quantitative analysis of the positive area for c-Fos, α-SMA, and Substance-P detected by immunohistochemistry. Data are expressed as means ± SD, *p < 0.05, **p < 0.01, ***p < 0.001, ****p < 0.0001, ns: no significance. n = 5 for all groups.

**FIGURE 5:**
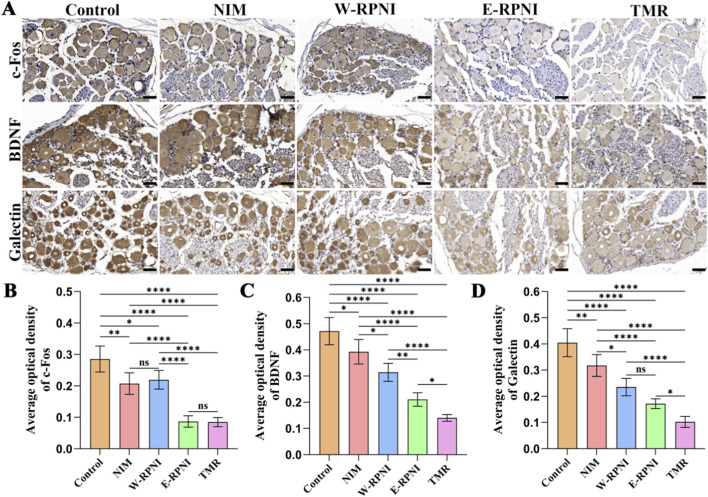
Immunohistochemical analysis of the ipsilateral DRG. **(A)** Immunohistochemical expression of c-Fos, BDNF, and Galectin in the DRG from each group. Scale bar = 40 μm. **(B–D)** Quantitative analysis of the average optical density values (IOD/Area) for c-Fos, BDNF, and Galectin. Data are expressed as means ± SD, *p < 0.05, **p < 0.01, ***p < 0.001, ****p < 0.0001, ns: no significance. n = 5 for all groups.

Substance-P is a sensory neuropeptide that mediates nociceptive transmission and neurogenic inflammation, and its expression is upregulated following nerve injury. c-Fos is an immediate early gene that serves as a marker of neuronal activation and heightened excitability within pain pathways (Chen et al., 2024; Ji et al., 2018). α-Smooth Muscle Actin (α-SMA) is a myofibroblast marker involved in the fibrotic encapsulation of injured nerves, potentially contributing to neuroma-associated pain both by directly inducing structural changes and by indicating localized mechanical irritation (Yan et al., 2012; Hillsley et al., 2022). These markers were examined to evaluate the inhibitory effect of TMR on neuroma-related pain (Figure 4A). Immunohistochemical analysis revealed that although the percentage of the c-Fos-positive area in the TMR group was not significantly different from that in the E-RPNI group, it remained the lowest among all groups (Figure 4B). For α-SMA and Substance P expression, the TMR group showed a significantly lower percentage of positive area than all other groups. Notably, for all three markers, the E-RPNI and W-RPNI groups demonstrated comparable expression levels, with no significant differences between them (Figures 4C,D). Furthermore, previous research has demonstrated that BDNF and members of the galectin family are persistently upregulated in the DRG following peripheral nerve injury, and are closely linked to ongoing axonal regeneration and pain sensitization (Pan et al., 2021). In our study, immunohistochemical analysis of the L4-L5 DRG showed that (Figure 5A) the average optical density of c-Fos expression in the TMR group followed a trend similar to that observed in the tibial nerve: it remained the lowest among all groups but did not differ significantly from the E-RPNI group (Figure 5B). Similarly, for both BDNF and Galectin, the TMR group exhibited significantly lower average optical densities than all other groups (Figures 5C,D), while E-RPNI and W-RPNI showed comparable expression levels.

### 3.4 Tibial nerve immunofluorescence evaluation

**FIGURE 6:**
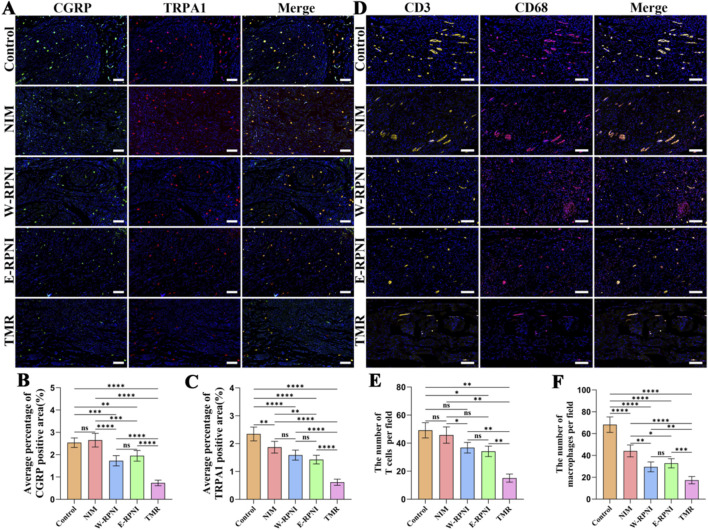
Immunofluorescence evaluation of the distal segment of the tibial nerve. **(A)** Immunofluorescence expression of CGRP and TRPA1 in longitudinal sections of the distal tibial nerve from each experimental group (scale bar = 100 µm). **(B,C)** Quantitative analysis of the immunopositive areas for CGRP and TRPA1. **(D)** Immunofluorescence expression of CD3, and CD68 in longitudinal sections of the distal tibial nerve from each experimental group (scale bar = 100 µm). **(E,F)** Statistical analysis of T lymphocytes (CD3) and macrophages (CD68) in each experimental group. Data are expressed as means ± SD, *p < 0.05, **p < 0.01, ***p < 0.001, ****p < 0.0001, ns: no significance. n = 5 for all groups.

Previous studies have identified TRPA1 and CGRP as critical mediators of mechanical allodynia and neuropathic pain following peripheral nerve injury (De Logu et al., 2022). TRPA1, a nociceptor channel expressed predominantly in peptidergic primary sensory neurons, plays a pivotal role in sustaining neuroinflammation and macrophage-dependent pain hypersensitivity. CGRP, a neuropeptide often co-expressed with TRPA1, can maintain increased excitability of primary sensory neurons, in part through TRPA1-mediated endosomal signaling, and its peripheral inhibition has demonstrated analgesic effects in models of neurogenic pain (Luo et al., 2025). Based on these mechanistic insights, we selected TRPA1 and CGRP as key molecular indicators to assess pain-related neuropathology in both the tibial nerve and the L4-L5 DRG in our model. In our study, immunofluorescence staining demonstrated that in tibial nerve sections, the TMR group exhibited the lowest expression levels of TRPA1 and CGRP among all experimental groups (Figure 6A). Quantitative analysis of fluorescence intensity confirmed that these reductions were statistically significant compared with the control, NIM, W-RPNI, and E-RPNI groups (Figures 6B,C). No significant differences were observed between the W-RPNI and E-RPNI groups for either TRPA1 or CGRP. These findings suggest that TMR intervention not only attenuates structural disorganization at the nerve stump, but may also mitigate mechanical allodynia via downregulation of TRPA1-CGRP-associated pain pathways.

Inflammatory cell infiltration was evaluated using CD68 (macrophage marker) and CD3 (T-cell marker) immunofluorescence in tibial nerve sections (Figure 6D). At 12 weeks post-surgery, the TMR group displayed markedly reduced numbers of CD68- and CD3-positive cells compared with all other groups, with statistically significant differences. Similarly, W-RPNI and E-RPNI groups showed comparable inflammatory cell counts with no significant difference (Figures 6E,F). These findings indicate that TMR effectively suppresses both neuroinflammatory mediators and immune cell accumulation at the neuroma site.

### 3.5 DRG immunofluorescence evaluation

**FIGURE 7:**
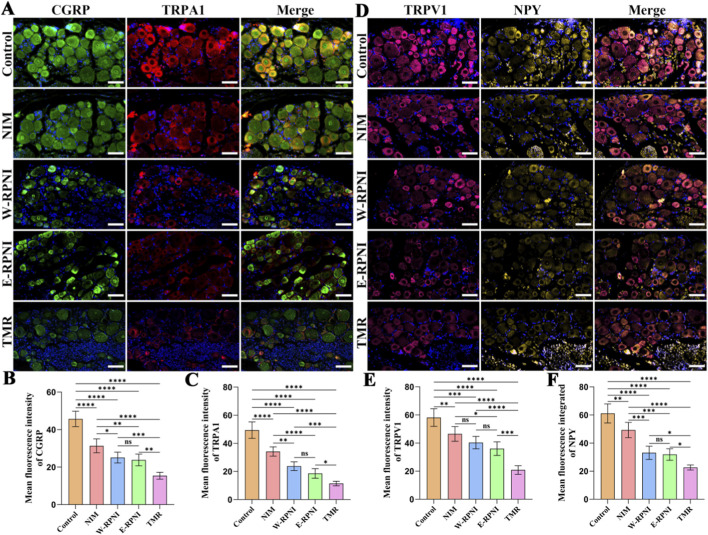
Immunofluorescence evaluation of the ipsilateral DRG. **(A)** Immunofluorescence expression of CGRP and TRPA1 in the DRG from each group (scale bar = 50 µm). **(B,C)** Quantitative analysis of the mean fluorescence intensity (MFI) for CGRP and TRPA1. **(D)** Immunofluorescence expression of TRPV1 and NPY in the DRG from each group (scale bar = 50 µm). **(E,F)** Quantitative analysis of the mean fluorescence intensity (MFI) for TRPV1 and NPY. Data are expressed as means ± SD, *p < 0.05, **p < 0.01, ***p < 0.001, ****p < 0.0001, ns: no significance. n = 5 for all groups.

TRPV1, a capsaicin-sensitive ion channel co-expressed in nociceptors, mediates thermal hyperalgesia and integrates inflammatory signals (Caterina and Julius, 2001), whereas NPY, a neuromodulator upregulated in DRG neurons after nerve injury, modulates pain hypersensitivity and neuronal excitability, potentially exacerbating allodynia through sympathetic-sensory coupling (Nelson et al., 2019). In addition to TRPV1 and NPY, CGRP and TRPA1 were also assessed in the DRG owing to their well-established involvement in neurogenic pain. Immunofluorescence staining revealed that the TMR group exhibited the lowest expression levels of CGRP and TRPA1 among all groups (Figure 7A), with markedly reduced fluorescence compared to the NIM and control groups. Quantitative analysis confirmed that these reductions were statistically significant relative to the control, NIM, W-RPNI, and E-RPNI groups for both markers (Figures 7B,C). Similarly, TRPV1 and NPY expression was lowest in the TMR group (Figure 7D), with significant differences compared to all other groups (Figures 7E,F). Notably, TRPV1 levels showed no significant difference between the NIM and W-RPNI groups. Across all four markers, no significant differences were observed between the W-RPNI and E-RPNI groups. These data indicate that TMR markedly downregulates multiple nociceptive pathways in the DRG, which may contribute to reduced central sensitization and alleviation of neuropathic pain behaviors.

### 3.6 qRT-PCR analysis

**FIGURE 8:**
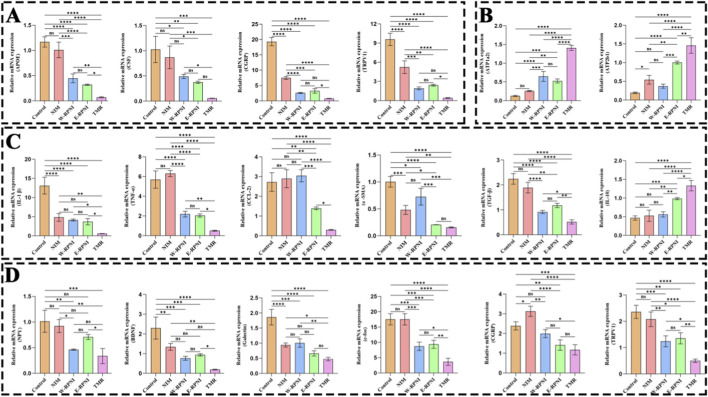
Relative expression of pain-, inflammation-, and fibrosis-related genes in the tibial nerve stump intervention area and ipsilateral DRG in each experimental group. **(A,B)** Relative mRNA expression of pain-related genes (APOE, CSF, CGRP, TRPV1, ATP1A2, ATP2B1) in the tibial nerve intervention area. **(C)** Relative mRNA expression of inflammation- and fibrosis- related genes (IL-1β, TNF-α, CCL-2, α-SMA, TGF-β, IL-10) in the tibial nerve intervention area. **(D)** Relative mRNA expression of pain-related genes (NPY, BDNF, CGRP, TRPV1, Galectin, cFos) in the corresponding DRG of the tibial nerve. Data are expressed as means ± SD,*p < 0.05, **p < 0.01, ***p < 0.001, ****p < 0.0001, ns: no significance. n = 3 for all groups.

Quantitative real-time PCR was performed to assess the relative mRNA expression of pain-, inflammation-, and fibrosis-related genes in both the tibial nerve intervention region and the corresponding L4-L5 DRG at 12 weeks postoperatively (Figure 8). For pain-related genes in the tibial nerve, expression levels of APOE, CSF, CGRP, and TRPV1 were markedly reduced in the TMR group compared with other groups, whereas the expression of the antinociceptive ion transport-related genes ATP1A2 and ATP2B1 was upregulated (Figures 8A,B). For inflammation- and fibrosis-associated markers, the TMR group exhibited significantly lower expression of the pro-inflammatory cytokines IL-1β, TNF-α, and CCL-2, as well as the fibrosis-related factors α-SMA and TGF-β, relative to all other groups (Figure 8C). In contrast, expression of the anti-inflammatory cytokine IL-10 was significantly elevated in the TMR group compared with other interventions. In the DRG, pain-related genes including NPY, BDNF, CGRP, TRPV1, Galectin, and c-Fos exhibited trends similar to those observed in the tibial nerve. The TMR group showed markedly reduced expression for all six targets, with statistically significant differences compared with other groups for all genes except c-Fos and CGRP. No significant differences in the expression of any of these DRG genes were detected between the E-RPNI and W-RPNI groups (Figure 8D).

Collectively, these transcriptional findings are consistent with the histological, immunohistochemical, and immunofluorescence results, indicating that TMR suppresses peripheral and central nociceptive signaling, attenuates inflammatory and fibrotic responses, and enhances the expression of antinociceptive modulators at the molecular level.

### 3.7 Pain-related protein expression in the DRG

Pain-Related Protein Expression in the DRG is a cluster of primary sensory neuron cell bodies located bilaterally in the intervertebral foramina, serving as the first relay station for peripheral sensory signals, including nociceptive input, to the central nervous system (Noh et al., 2020). Following peripheral nerve injury, DRG neurons and associated satellite glial cells undergo pronounced molecular and functional changes that contribute to nociceptive signal transmission, maintenance of neuropathic pain, and central sensitization (Yuan et al., 2020). Therefore, assessing pain-related protein expression in the DRG provides valuable molecular insight into the efficacy of different interventions in modulating neuroma-associated pain.

**FIGURE 9:**
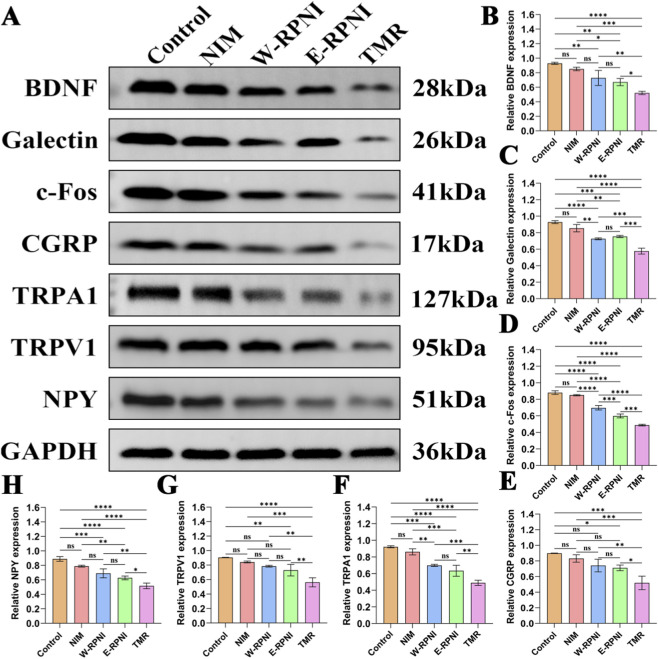
Western blot analysis of the ipsilateral DRG at 12 weeks postoperatively in each experimental group. **(A)** Western blot results for BDNF, Galectin, c-Fos, CGRP, TRPA1, TRPV1, and NPY . **(B–H)** Statistical analysis of the relative expression levels of BDNF, Galectin, c-Fos, CGRP, TRPA1, TRPV1, and NPY, normalized to GAPDH. Data are expressed as means ± SD, *p < 0.05, **p < 0.01, ***p < 0.001, ****p < 0.0001, ns: no significance. n = 3 for all groups.

To further assess the inhibitory effects of each intervention on pain during neuroma development, we quantified the protein expression of pain-related markers in the L4-L5 DRG corresponding to the injured tibial nerve using Western blot analysis (Figure 9A). At 12 weeks postoperatively, the expression levels of BDNF, Galectin, c-Fos, CGRP, TRPA1, TRPV1, and NPY varied among groups; notably, the TMR group consistently exhibited the lowest levels, with statistically significant reductions compared with all other groups (Figures 9B–G). For all proteins except c-Fos, no significant differences were observed between the W-RPNI and E-RPNI groups.

## 4 Discussion

In this study we investigated whether targeted muscle reinnervation (TMR) can prevent painful neuroma formation and attenuate neuropathic pain in a rat tibial nerve transection model. By comparing TMR with nerve-into-muscle coaptation (NIM) and two variants of regenerative peripheral nerve interface-wrapped RPNI and embedded RPNI (W-RPNI and E-RPNI)-we sought to determine which technique most effectively restores physiological innervation, alleviates pain behaviours, and modulates the inflammatory and nociceptive milieu at the injured nerve stump. Our data show that immediate TMR results in the most robust recovery across behavioural, histological and molecular measures. Rats receiving TMR exhibited superior gait metrics (extended stand duration and larger contact area), and sensory hypersensitivity showed a marked reversal toward baseline levels; neuroma tenderness was also minimized in this group. Histologically, TMR stumps demonstrated comparatively more organized neural architecture with reduced fibrotic encapsulation and collagen deposition relative to controls. While improvements in behavioral and gait outcomes were observed in the W-RPNI and E-RPNI groups, differences remained in their fiber organization consistency and collagen deposition profiles compared to the TMR group. Overall, within this rat tibial nerve transection model, these findings suggest that TMR provided consistent mitigation of neuroma-associated pain behaviors and fibrotic remodeling among the strategies tested, supporting the concept that directing regenerating axons into a denervated motor pathway may facilitate more orderly reinnervation.

Our behavioral findings demonstrate that, compared with NIM, W-RPNI, and E-RPNI, TMR produces superior functional recovery. In the TMR group, stand-phase duration and maximal contact area of the affected limb improved progressively and surpassed the gains achieved with RPNI variants by week 12. Mechanical hypersensitivity and thermal hyperalgesia were alleviated earlier and more effectively after TMR compared with other interventions. These results build upon prior clinical and experimental studies, reinforcing that early TMR prevents the onset of neuropathic pain behaviors and can even reverse established hypersensitivity when performed within 3 weeks of nerve injury (Roth et al., 2023). In a sciatic nerve injury model, Roth et al. demonstrated that immediate or 3-week TMR prevented mechanical and thermal hypersensitivity, whereas delaying the procedure to 12 weeks failed to reverse pain behaviors (Roth et al., 2023). Using a tibial nerve transection model, we similarly observed that immediate TMR prevented declines in withdrawal thresholds. Although recent direct comparisons between TMR and RPNI reported analgesic benefits for both approaches at 10 weeks post-transection (Senger et al., 2023), our data show that TMR provided faster and more pronounced pain relief. Clinically, acute TMR performed at the time of amputation reduces symptomatic neuroma recurrence and pain interference scores (Goodyear et al., 2024), and a randomized trial confirmed improved phantom limb pain outcomes compared with standard neuroma excision (Dumanian et al., 2019). Furthermore, a recent systematic review and meta-analysis (11 studies) found that TMR was associated with lower phantom limb pain/residual limb pain scores, better functional outcomes, and reduced opioid use versus standard nerve management (Zimbulis et al., 2024). In contrast, Clinical evidence suggests that RPNI improves neuroma-related residual limb pain, whereas improvements in phantom limb pain are often modest and PLP may persist in a substantial proportion of patients, potentially reflecting ongoing central sensitization (Lee et al., 2025; Kubiak et al., 2022). Collectively, these findings, together with our preclinical data, suggest that implanting a nerve into a muscle graft without achieving reinnervation of a functional motor end-plate fails to adequately guide axonal regeneration or suppress aberrant sprouting.

Histological analyses provide insight into the superior outcomes observed with TMR. In controls, distal nerve stumps consistently formed bulbous neuromas with disorganized axonal regeneration, muscle infiltration, and dense collagen deposition. TMR markedly reduced pathological fibrosis, maintained orderly regeneration at the coaptation site, and minimized collagen accumulation. Sirius red staining revealed that type I and type III collagen levels were lowest after TMR, even compared with RPNI variants that also reduce collagen deposition. Pathological fibrosis is a recognized driver of neuroma-associated pain, as entrapped axons are exposed to chronic mechanical tension and stimulation. A review of tissue-engineered treatments for traumatic neuromas reported dense α-SMA-positive myofibroblast accumulation within neuromas, where they secrete extracellular matrix and contractile proteins correlated with pain severity (Wan et al., 2024). That review also noted CGRP expression in neuroma axons, which activates macrophages to release IL-6, promoting fibrosis and nociceptor sensitization (Wan et al., 2024). Our histological and molecular findings align with these observations: α-SMA expression was lowest in the TMR group, collagen deposition was minimal, and CGRP was reduced at both the nerve stump and DRG. Moreover, reduced TGF-β1 expression in TMR tissue indicates a potential inhibitory tendency on the differentiation of fibroblasts into contractile myofibroblasts.

Immunohistochemistry further underscores that TMR attenuates nociceptive signaling. Substance P and c-Fos were significantly reduced in the TMR group (Miao et al., 2022), with TMR uniquely decreasing both Substance P and α-SMA, whereas W-RPNI and E-RPNI reduced c-Fos alone. Although reductions in individual neuropeptides may accompany analgesia, double-knockout mice lacking both Substance P and CGRPα exhibit intact nociceptive responses (Zhang et al., 2025), indicating that TMR exerts its benefit through broad suppression of aberrant axonal sprouting, neuropeptide release, and inflammatory signaling rather than elimination of a single pathway.

BDNF is another mediator linking peripheral nerve injury to central sensitization; elevated levels are associated with greater pain severity and hypersensitivity (Thakkar and Acevedo, 2023). Galectin-3 acts as a microglial activator and contributes to postoperative hyperalgesia (Luo and Huang, 2024). Immunostaining of L4–L5 DRG demonstrated that TMR produced the lowest BDNF and Galectin-3 optical densities. Peripheral nerve injury can upregulate BDNF in uninjured DRG neurons, and intrathecal BDNF neutralization attenuates thermal hyperalgesia (Fukuoka et al., 2001). Galectin-3 upregulation after spinal nerve ligation promotes pro-inflammatory cytokine release and pain (Ma et al., 2016). Elevated serum BDNF has also been proposed as a biomarker of chronic pain severity in older adults (Miao et al., 2022). Inhibiting both Galectin-3 and BDNF may restrict spinal microglial activation and synaptic remodeling, reducing the risk of chronic pain. Pro-inflammatory microglia release TNF-α, IL-1β, and IL-6 to drive sensitization, whereas M2-polarized microglia secrete IL-10 and TGF-β to promote repair and analgesia (Sideris-Lampretsas et al., 2023). Taken together, these results suggest that TMR’s analgesia is mediated via multiple mechanisms: directing regenerating axons into motor targets, reducing local fibrosis and inflammation, downregulating ion channel and neuropeptide expression, and dampening microglial and neurotrophic signaling to prevent central sensitization.

Immunofluorescence provided further molecular insights. In the tibial nerve, TMR reduced TRPA1 and CGRP more effectively than RPNI. In the DRG, TMR decreased TRPA1, CGRP, TRPV1, and NPY. TRPA1 and CGRP are co-expressed in peptidergic nociceptors contributing to mechanical allodynia and neurogenic inflammation; TRPA1 activation enhances excitability and CGRP release, while its blockade reduces hypersensitivity (Masood et al., 2023). TRPV1 mediates thermal hyperalgesia and is implicated in chemotherapy-induced neuropathic pain, with inhibition alleviating mechanical hypersensitivity (Zimbulis et al., 2024). NPY acts via Y1 receptor-expressing neurons to inhibit chronic inflammatory and neuropathic pain, and exogenous NPY or Y1 agonists provide analgesia (Nie and Taylor, 2025). However, NPY upregulation after injury may facilitate sympathetic–sensory coupling and ectopic firing. Suppression of TRPV1 and NPY thus may help restore the balance between pro- and anti-nociceptive neuropeptides (Zimbulis et al., 2024).

qPCR confirmed that TMR downregulated pain-related genes (APOE, CSF, CGRP, TRPV1) and upregulated antinociceptive ion transporters ATP1A2 and ATP2B1. Known inflammatory mediators IL-1β, TNF-α, CCL-2, and TGF-β were suppressed, while IL-10 was markedly increased (Wan et al., 2024). In enriched environment models, reduced CCL2 and increased IL-10 are linked to enhanced regeneration and pain relief (Luo and Huang, 2024). IL-10 drives macrophage and microglial polarization toward an M2 phenotype, fostering anti-inflammatory cytokine release and inhibiting TNF-α and IL-1β (Garcia-Dominguez, 2025). This immune shift likely contributes to the reduced CD68^+^ macrophage and CD3^+^ T-cell infiltration seen with TMR. Conversely, sustained TNF-α, IL-1β, and CCL-2 levels in RPNI and NIM maintain a pro-inflammatory environment that sensitizes nociceptors and perpetuates pain (Ortola et al., 2025). Although W-RPNI and E-RPNI yielded intermediate reductions in inflammatory markers, they did not enhance ATP1A2 or ATP2B1 expression, which supports neuronal membrane potential restoration and Ca^2+^ clearance.

We observed that TMR markedly reduced immune-cell infiltration at the nerve stump. CD68 and CD3 staining revealed that macrophages and T cells were lowest in TMR, with higher counts in W-RPNI and E-RPNI. By redirecting regenerating axons to functional motor targets, TMR likely minimizes immune activation at the stump. Evidence from a prior animal study has indicated that delaying TMR by 12 weeks abolishes its analgesic benefit, whereas TMR performed acutely or within a short 3-week interval can effectively prevent or reverse pain behaviors to baseline levels (Roth et al., 2023). Our findings with immediate TMR further support the efficacy of early intervention in directing axonal regrowth. While this study focuses on the immediate application of TMR, it is clinically recognized that other surgical strategies, such as regenerative peripheral nerve interfaces (RPNI), also serve as effective options for managing amputation-related pain (Senger et al., 2023). These collective findings underscore the importance of timely intervention to provide an appropriate physiological target for regenerating axons and potentially mitigate maladaptive plasticity.

This study has several limitations. Only male rats were used, and outcomes were evaluated over a twelve-week period, leaving sex differences and long-term effects unresolved. Although a range of behavioral tests was applied, they cannot fully capture spontaneous pain behaviors. In addition, molecular analyses were performed at a single time point, which may not reflect dynamic changes during regeneration. Future studies should extend the observation period, include both sexes, and explore whether combining TMR with biomaterial conduits or pharmacological modulation of TRP channels and neuropeptides can enhance therapeutic efficacy.

## 5 Conclusion

In summary, TMR most effectively prevented neuroma formation and alleviated neuropathic pain compared with W-RPNI, E-RPNI, and NIM in a rat tibial nerve transection model. It achieved superior functional recovery, minimized fibrosis, and showed the most favorable molecular profile by suppressing pain- and inflammation-related markers while upregulating antinociceptive and anti-inflammatory genes. In our experimental setup, RPNI and NIM exhibited varying degrees of efficacy, whereas TMR consistently provided more comprehensive advantages across the parameters tested. These findings establish TMR as a highly promising surgical strategy tested in this model and support clinical translation.

## Data Availability

The original contributions presented in the study are included in the article/Supplementary Material, further inquiries can be directed to the corresponding authors.
