# Supplementary material for: Targeted muscle reinnervation attenuates neuropathic pain and neuroma development in a rat model of tibial nerve transection
Source: Front Bioeng Biotechnol. 2026 Feb 27;14:1758496. doi: 10.3389/fbioe.2026.1758496 (PMC12983234; doi:10.3389/fbioe.2026.1758496)
Supplement: Supplementary file 1 [file Supplementaryfile1.docx]

Supplementary Material

# 1 Supplementary Table

| ****Table S1.** Sequences of primers used for quantitative real-time PCR.** | | |
| --- | --- | --- |
| Target Gene | Forward sequence | Reward sequence |
| APOE | **CTTCTGGGATTACCTGCG** | **TGCCTTTACTTCCGTCATAG** |
| CSF | **CGAAAAGAACGAAGACGTAG** | **CTTGTATAGCTTCAGGCGG** |
| TRPV1 | **CTTCACTACCAGGAGTCGTA** | **GAGCTGACAGTGATGATAGG** |
| CGRP | **GACCTCAACAAGTTTCACAC** | **TAGTTGCCAAAATAGGGGTG** |
| ATP2b1 | **AATTGACGAGAGTTCGTTGA** | **AGTACCTGAAAGAAGCAAGG** |
| ATP1a2 | **TTTTTGGGGGCTTCTCTATC** | **ATTGTCATTGGATGGTTCGT** |
| CCL2 | **AACCAAGTGAGATCAGAAACT** | **GAGTGGATGCATTAGCTTCA** |
| α-SMA | **CTTCCAGCCATCTTTCATTG** | **GACGTTGTTAGCATAGAGGT** |
| IL-1β | **GAGTGTGGATCCCAAACAAT** | **TCCACTTTGGTCTTGACTTC** |
| TNF-α | **AGATGGGCTGTACCTTATCTA** | **GTATGAAATGGCAAATCGGC** |
| TGF-β | **ACCGACATCTCAGAGTTCATC** | **AAGGACGCTGGCTGTAAT** |
| IL-10 | **TTTAATAAGCTCCAAGACAAAGG** | **TTCATTTTGAGTGTCACGTAG** |
| Bdnf | **ACGAGGAAGGGTGTTCCAC** | **CGTTTGCTTCTTTCATGGGC** |
| c-Fos | **ACAGCCTTTCCTACTACCAT** | **TGGGGATAAAGTTGGCACTA** |
| Galectin | **AATTCAAATTCCCCAACCG** | **GCCACACACTTAATCTTGAA** |
| NPY | **TCTCATCACCAGACAGAGAT** | **GGCATTTTCTGTGCTTTCTC** |
| GAPDH | **GCCATCACTGCCACTCAGAAGAC** | **ATGACCTTGCCCACAGCCTTG** |

## 2 Supplementary Figure


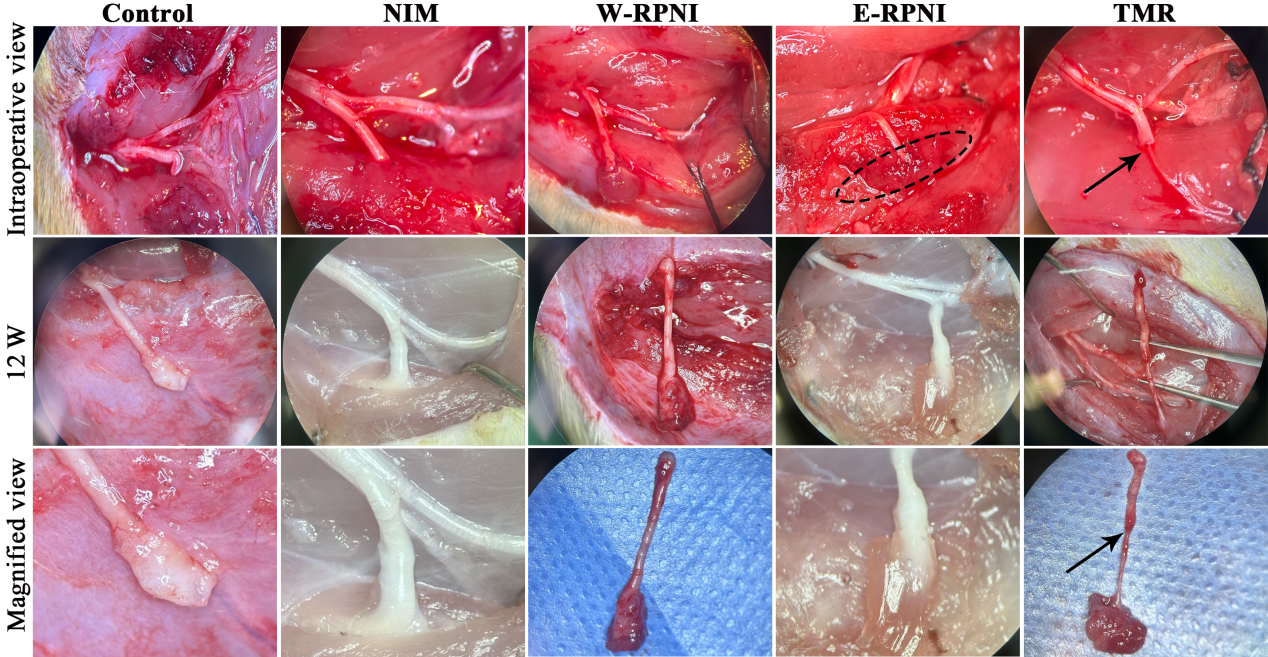


**Supplementary Figure 1.** Intraoperative views of the tibial nerve at the time of model establishment and at 12 weeks postoperatively in the Control, NIM, W‑RPNI, E‑RPNI, and TMR groups. The third row shows magnified intraoperative images of each group at 12 weeks after intervention by the five different surgical procedures. The oval dashed line indicates the portion of the contralateral extensor digitorum longus muscle transplanted into the belly of the biceps femoris muscle, and the black arrow points to the site of coaptation between the tibial nerve and the motor branch innervating the biceps femoris muscle.
